# Treatment Outcomes for Extensively Drug-Resistant Tuberculosis and HIV Co-infection

**DOI:** 10.3201/eid1903.120998

**Published:** 2013-03

**Authors:** Max R. O’Donnell, Nesri Padayatchi, Charlotte Kvasnovsky, Lise Werner, Iqbal Master, C. Robert Horsburgh

**Affiliations:** Author affiliations: Albert Einstein College of Medicine, Bronx, New York, USA (M.R. O’Donnell);; Centre for AIDS Programme of Research in South Africa, Durban, South Africa (M.R. O’Donnell, N. Padayatchi, L. Werner);; University of Maryland School of Medicine, Baltimore, Maryland, USA (C. Kvasnovsky);; King George V Hospital, Sydenham, South Africa (I. Master);; Boston University School of Public Health, Boston, Massachusetts, USA (C.R. Horsburgh, Jr.);; Boston University School of Medicine, Boston (C.R. Horsburgh, Jr.)

**Keywords:** tuberculosis and other mycobacteria, bacteria, extensively drug-resistant tuberculosis, XDR TB, HIV, viruses, co-infection, treatment outcomes, South Africa

## Abstract

High mortality rates have been reported for patients co-infected with extensively drug-resistant tuberculosis (XDR-TB) and HIV, but treatment outcomes have not been reported. We report treatment outcomes for adult XDR TB patients in KwaZulu-Natal Province, South Africa. Initial data were obtained retrospectively, and outcomes were obtained prospectively during 24 months of treatment. A total of 114 XDR TB patients were treated (median 6 drugs, range 3–9 drugs); 82 (73%) were HIV positive and 50 (61%) were receiving antiretroviral therapy. After receiving treatment for 24 months, 48 (42%) of 114 patients died, 25 (22%) were cured or successfully completed treatment, 19 (17%) withdrew from the study, and 22 (19%) showed treatment failure. A higher number of deaths occurred among HIV-positive patients not receiving antiretroviral therapy and among patients who did not show sputum culture conversion. Culture conversion was a major predictor of survival but was poorly predictive (51%) of successful treatment outcome.

## Abstract 2

Sputum culture conversion was poorly predictive of successful treatment.

Drug-resistant tuberculosis (TB) is a critical threat to TB control and global public health (*1*–*3*). Nowhere is this threat more pressing than in South Africa, where drug-resistant TB and HIV have converged in a deadly syndemic defined by increased incidences of TB and HIV (*4*), endemic transmission of drug-resistant TB strains (*5*), high mortality rates (*6*), and poor treatment outcomes (*7*). The most drug-resistant form of TB, extensively drug-resistant tuberculosis (XDR TB) (*8*) has been reported in 70 countries and comprises an increasing proportion of drug-resistant TB cases (*1*).

The global epicenter of the XDR TB and HIV syndemic is KwaZulu-Natal Province in South Africa, where nearly 400 XDR TB patients, 70% co-infected with HIV, were admitted to a provincial TB referral hospital for initiation of therapy during 2003–2008 (*9*). To contextualize this incidence, 73% (573/782) of all XDR TB cases reported to the World Health Organization globally during 2002–2009 were from South Africa (*3*,*10*). It is estimated that 50% of patients with a diagnosis of XDR TB in KwaZulu-Natal Province do not survive to treatment referral (*11*). Therefore, hospital-based surveillance represents a major underestimate of cases of co-infection with XDR TB and HIV in the province.

Without adequate second-line TB and HIV treatment, reported mortality rates for persons co-infected with XDR TB and HIV approach 100% (*6*). Among XDR TB patients who survive to initiation of second-line TB therapy, early treatment outcomes reported by our group and others describe low rates of sputum culture conversion, major adverse events, and a substantial number of early deaths (*12**–**14*). To our knowledge there are no published reports of outcomes for patients co-infected with XDR TB and HIV at the end of TB treatment. This report describes treatment outcomes, adverse events, and risk factors for death among patients in South Africa with XDR TB, most of whom were co-infected with HIV.

## Methods

### Study Participants

Early culture conversion and mortality rate data for the first 12 months after initiation of treatment for XDR TB in the first 60 patients in this cohort have been reported (*12*). In brief, patients were adult (>18 years of age) XDR TB patients consecutively admitted to a public TB referral hospital in KwaZulu-Natal Province in South Africa during December 1, 2006–October 31, 2007 for initiation of treatment for XDR TB. Patients with complications and drug-resistant TB are referred to this facility at the discretion of the treating physician and are admitted depending on patient acuity and bed availability. The practice during the study period was to admit all XDR TB patients for initiation of second-line TB treatment. Eligible participants had culture-proven TB and *Mycobacterium tuberculosis* infection, and drug susceptibility testing results meeting the revised World Health Organization criteria for XDR TB (*10*). In addition, patients agreed to begin appropriate second-line and third-line anti-TB treatment. All anti-TB treatment regimens were determined by treating physicians on the basis of drug susceptibility results and adverse drug reactions. All treatment was provided through the South African public health system and directly observed therapy was provided. However, we did not assess the quality of directly observed therapy support or adherence.

### Study Design

Patients who met eligibility criteria were identified retrospectively, and information was collected by chart review and review of an electronic laboratory database. Information on demographics, risk factors and adverse drug reactions, and treatment outcomes were collected retrospectively. Treatment outcomes of enrolled patients were followed through December 31, 2009, to ensure that each patient had ≥24-months of follow-up. Standard drug-resistant TB treatment outcome definitions were used to define outcome (*15*). Treatment outcomes were cure, treatment completion, death, and treatment default (*15*).

All drug-resistant TB treatment outcomes were mutually exclusive and were defined at 24 months except in the case of treatment default and death, which were defined when they occurred. Cure was defined as treatment for 24 months and ≥5 consecutive negative culture results in the final 12 months of treatment. Treatment completion was defined as treatment for 24 months, with clinical improvement, and negative cultures after treatment, but did not meet the definition for cure because of lack of bacteriologic results (<5 cultures performed in the final 12 months of therapy). If 1 culture was positive for TB but there was no clinical deterioration, the patient was still considered cured or that treatment was completed provided that this result was followed by >3 monthly negative cultures. Treatment failure was defined as treatment for 24 months but with ≥2 of 5 cultures in the final 12 months positive, or if any 1 of the final 3 cultures was positive for TB, or if clinical failure was indicated by the clinician. Default was defined as treatment interrupted for ≥2 consecutive months for any reason. Death was defined as death of a patient for any reason during treatment.

TB sputum culture conversion was defined as having ≥2 negative consecutive sputum cultures 30 days apart after initiation of treatment. Patients may have subsequently showed reversion to a status of TB sputum culture positive. Adverse events were recorded by clinical staff. Severe adverse events were defined as events causing new hospitalization, stopping a drug in the regimen, urgent/emergent treatment, or death (*16*). In addition, electrolyte abnormalities (potassium level <2.5 mmol/L or magnesium level <1.5 mmol/L) attributed to medication use, were considered to be severe adverse events. The study protocol was approved by the Biomedical Research Ethics Committee of the University of KwaZulu-Natal and the Institutional Review Board of Boston University Medical Center.

### Drug Susceptibility Testing

Drug susceptibility testing for first-line and second-line drugs was performed at the provincial TB referral laboratory in Durban, South Africa. Culture positivity was determined by using the BACTEC MGIT 960 fluorometric system (Becton Dickinson Diagnostics, Sparks, MD, USA). Drug susceptibility testing for isoniazid, rifampin, ethambutol, streptomycin, ethionamide, ofloxacin, and kanamycin was performed by using the modified proportional growth method on 7H11 agar according to standard techniques (*17*,*18*). Drug susceptibility testing for capreomycin, *p*-aminosalacylic acid (PAS), terizidone, cycloserine, and pyrazinamide was not available during the study period because of technical and resource availability issues. Sputum samples for culture were routinely obtained on a monthly basis.

### Statistical Analysis

All participants in the study were included in an analysis of risk factors for survival and unfavorable treatment outcome. For the survival analysis, we included all patients who died, even if they defaulted before death, as deaths. For the unfavorable treatment outcome analysis, unfavorable treatment outcome included death, treatment failure, and default. Successful treatment outcome included cure and treatment completion. Cox proportional hazards models were used to estimate hazard ratios (HRs) and 95% CIs. Significant variables or variables that caused >10% change in the bivariate HR were included in the multivariate model. We calculated 95% CIs by using a normal approximation of the binomial distribution. The Fischer exact test or χ^2^ test was used to compare categorical variables. Medians were compared by using the Wilcoxon Mann-Whitney U test. Kaplan-Meier survival curves for death and for time to culture conversion were calculated from time of XDR TB treatment initiation with appropriate anti-TB drugs. Participants were censored at time of default or death. The p values for survival analysis were determined by using Cox proportional hazards model adjusted for potential confounding variables, as per above. Analysis was performed by using SAS version 9.2 software (SAS Institute, Cary, NC, USA).

## Results

During 2006–2007, a total of 6,127 persons in KwaZulu-Natal Province were given a diagnosis of multidrug-resistant TB (MDR TB) (5,612) or XDR TB (536) (*19*). Of those 6,127 persons, 2,013 (1,771 with MDR-TB and 242 with XDR TB) were admitted to King George V Hospital for initiation of treatment during the 2-year study period (December 2006–October 2007). During this period, 130 consecutive patients with XDR TB were admitted to King George V Hospital for initiation of treatment. Four of these patients refused treatment, 2 patients had insufficient data, 6 patients died before the start of treatment, and 4 patients were <18 years of age. The remaining 114 XDR TB patients were eligible and were analyzed during the study.

The patients included in this study were transferred from 41 distinct municipalities or areas representing 9 (82%) of 11 health districts in KwaZulu-Natal Province. The most common addresses patients reported were in the Tugela Ferry catchment area (31%), metropolitan Durban (14%), and Pietermaritzburg (11%). Most (57%) patients were female patients, young (median age 35 years), and co-infected with HIV (77% with a known test result) (Table 1). Female patients were significantly younger (median age 31 years vs. 39 years; p<0.001) and more likely to be co-infected with HIV (80% vs. 61%; p = 0.01) than male XDR TB patients. Most (81%) patients had been treated previously for TB; fewer (37%) had been previously treated for MDR-TB. Fifty (61%) of 82 patients with known HIV infection were receiving antiretroviral therapy (ART) before hospital admission or initiated ART therapy early during treatment for XDR TB with efavirenz-based regimens. A total of 42 (37%) patients showed TB sputum culture conversion during treatment. Eighteen (43%) of 42 patients who showed culture conversion subsequently showed culture reversion (n = 7), defaulted (n = 7), or died (n = 4).

**Table 1:** Demographic characteristics of patients with XDR TB, KwaZulu-Natal Province, South Africa*

Characteristic	All patients, n = 114	Female patients, n = 65	Male patients, n = 49	p value
Sex				
M	49 (43.0)	NA	NA	NA
F	65 (57.0)	NA	NA	NA
Age, y				
18–25	16 (14.0)	16 (24.6)	0	<0.0001
26–35	42 (36.8)	27 (41.5)	15 (30.6)	<0.0001
36–50	46 (40.4)	21 (32.3)	25 (51.0)	NA
>50	10 (8.8)	1 (1.5)	9 (18.4)	MA
Median age (IQR)	35 (30–42)	31 (26–37)	39 (35–47)	NA
HIV status				
Positive	82 (71.9)	52 (80.0)	30 (61.2)	0.0153
Negative	25 (21.9)	8 (12.3)	17 (34.7)	NA
Unknown	7 (6.1)	5 (7.7)	2 (4.1)	NA
CD4 cell count/mm^3^†				
Known	55 (67.1)	38 (73.1)	17 (56.7)	0.1487
Not determined	27 (32.9)	14 (26.9)	13 (43.3)	0.1426
Median (IQR)	197 (80–300)	222 (71–316)	130 (83–254)	NA
Receiving ART†				
Yes	50 (61.0)	34 (65.4)	16 (53.3)	0.3492
No	32 (39.0)	18 (34.6)	14 (46.7)	NA
Severe adverse event‡				
Yes	29 (25.4)	29 (25.4)	12 (24.5)	1.0000
No	85 (74.6)	48 (73.9)	37 (75.5)	NA
Previous TB treatment				
Yes	92 (80.7)	53 (81.5)	39 (79.6)	0.7216
No	15 (13.2)	9 (13.9)	6 (12.2)	NA
Unknown	7 (6.1)	3 (4.6)	4 (8.2)	NA
Previous MDR TB diagnosis				
Yes	69 (60.5)	41 (63.1)	28 (57.1)	0.5649
No	45 (39.5)	24 (36.9)	21 (42.9)	NA
Health care worker				
Yes	6 (5.3)	4 (6.2)	2 (4.1)	0.6982
No	108 (94.7)	61 (93.9)	47 (95.9)	NA
Type of TB				
Pulmonary	103 (90.4)	58 (89.2)	45 (91.8)	NA
Extrapulmonary	11 (9.7)	7 (10.8)	4 (8.2)	NA
Culture conversion, mo§				
None	72 (63.2)	40 (61.5)	32 (65.3)	0.2523
<2	16 (14.0)	7 (10.8)	9 (18.4)	NA
>2	26 (22.8)	18 (27.7)	8 (16.3)	NA

*Values are no. (%) unless otherwise indicated. XDR TB, extensively drug-resistant tuberculosis; NA, not applicable; IQR, interquartile range; ART, antiretroviral therapy; MDR TB, multidrug-resistant TB.  †Among HIV-positive patients only.  ‡Resulted in changes in clinical status or electrolyte abnormalities or required change of TB treatment regimen. §After initiation of treatment.

Patients were treated with a median of 6 drugs (interquartile range [IQR] 5–7 drugs). The most common initial regimens included capreomycin (90%), PAS (90%), pyrazinamide (92%), ethionamide (92%), ethambutol (97%), cycloserine (66%), or terizidone (30%). Moxifloxacin (1%) and leveofloxacin (0%) were not available through the public health care system during the study period.

Adverse events during treatment occurred in 58% of patients; severe adverse events occurred in 25% of patients and were not associated with HIV status, ART, or treatment default. Within the HIV co-infected subgroup, there was an association between adverse events and death, this association was not significant by multivariate analysis. Physicians infrequently recorded a specific drug associated with the adverse event (29%): cycloserine (12%), capreomycin (8%), and PAS (4%) were the most common drugs related to adverse events. There were 8 episodes of psychosis or severe psychiatric illness attributed to cycloserine, which resulted incessation of the use of this drug. There were 4 deaths in the cohort attributed to hypokalemia or hypomagnesemia related to use of capremoycin. These events decreased over the study period as physicians empirically supplemented potassium and magnesium for patients during treatment with capreomycin.

**Figure 1:**
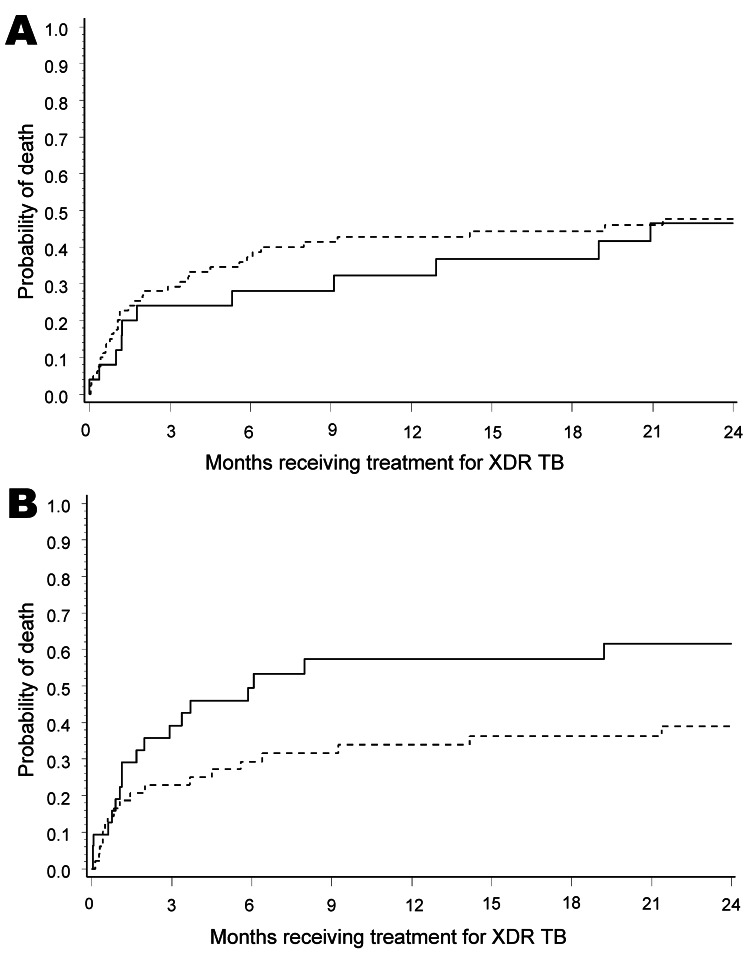
Kaplan-Meier curves for A) 114 HIV-positive (dashed line) and HIV-negative (solid line) patients receiving treatment for extensively drug-resistant tuberculosis (XDR TB) (p = 0.4966); and B) 82 HIV-infected patients with XDR TB receiving (dashed line) and not receiving (solid line) antiretroviral therapy (p = 0.0330), KwaZulu-Natal Province, South Africa. p values were adjusted for sex, TB treatment history, and HIV status.

**Figure 2:**
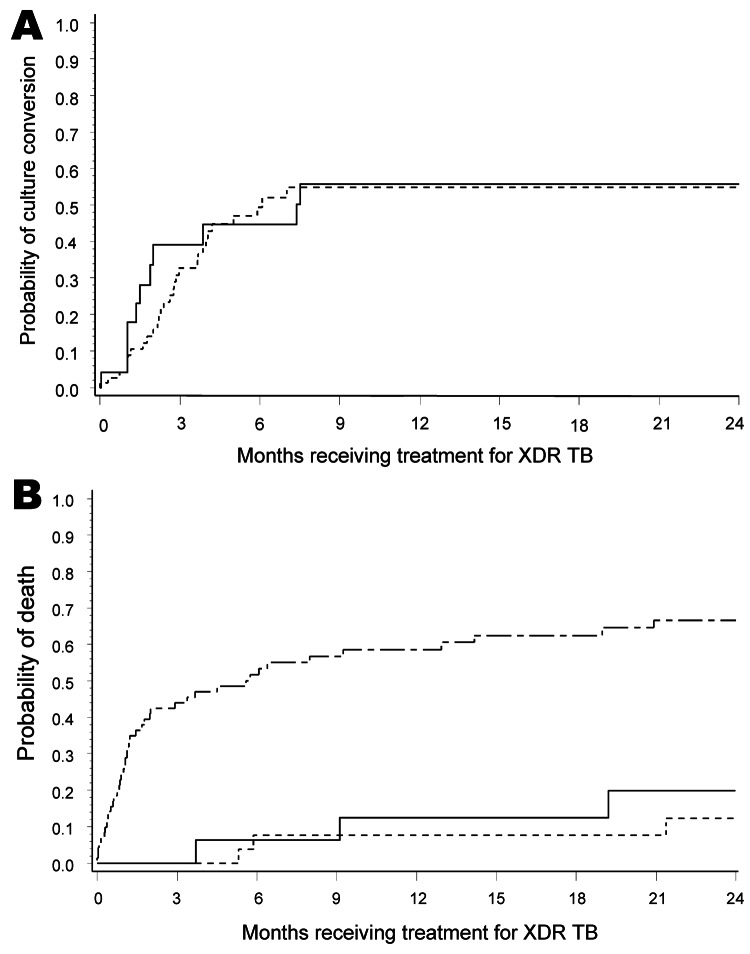
A) Kaplan-Meier curves for sputum culture conversion for HIV-positive (dashed line) and HIV-negative (solid line) patients with extensively drug-resistant tuberculosis (XDR TB) receiving treatment, KwaZulu-Natal Province, South Africa. Sputum culture conversion is defined as 2 consecutive monthly TB cultures with no growth after 6 weeks of incubation after initiation of treatment (p = 0.706). p value was adjusted for age, initial smear result, and HIV status. B) Kaplan-Meier curves for patients receiving treatment for XDR TB stratified by sputum culture conversion status (p<0.0001). Solid line indicates conversion ≤2 months after initiation of treatment, dashed line indicates conversion >2 months after initiation of treatment, and top line with small and large dashes indicates no conversion. p values were adjusted for sex, TB treatment history, and HIV status. There was no significant difference between patients who showed culture conversion <2 months and >2 months after initiation of treatment (p = 0.5182).

Treatment outcomes were determined 24 months after the initiation of treatment (Table 2). All treatment outcome categories were mutually exclusive. By 24 months, 48 (42%) of 114 patients had died, 25 (22%) of 114 were either cured or completed treatment, 19 (17%) of 114 defaulted, and 22 (19%) of 114 showed treatment failure (Table 2). Kaplan-Meier survival and culture conversion curves from start of XDR TB therapy are shown in Figures 1 and 2. Deaths of patients after they defaulted (n = 1) were counted as deaths in survival analysis. HIV status was not associated with a higher mortality rate or culture conversion, but among HIV-infected XDR TB patients, receiving ART was associated with improved survival but not improved sputum culture conversion. Patients who showed culture conversion early or late during treatment had improved survival by Kaplan-Meier analysis (Figure 2, panel B).

**Table 2:** Twenty-four month treatment outcomes for 114 patients with extensively drug-resistant tuberculosis, KwaZulu-Natal Province, South Africa*

Treatment outcome	No. (%) patients
Favorable	
Cure	15 (3.2)
Completed	10 (8.8)
Unfavorable	
Withdrew*	19 (16.7)
Failure	22 (19.3)
Died	48 (42.0)

*One patient initially defaulted and subsequently died.

TB culture conversion at 2 months of treatment was associated with survival by bivariate analysis (HR 5.55, 95% CI 1.75–20.0) and multivariate analysis (HR 5.0, 95% CI 1.49–16.67). Sputum culture conversion was not included as a variable in Table 3 because it is an intermediate in the causal pathway. Among all XDR TB patients, none of the included variables were associated with death (Table 3) or unfavorable treatment outcome as a composite outcome of death, treatment failure, and default. Among the subset of HIV co-infected XDR TB patients (Table 4), ART was protective against death by multivariate analysis (HR 0.46, 95% CI 0.22–0.94). When further stratified by CD 4 T-cell count/mm^3^, HIV co-infected XDR TB patients with CD4 cell counts >200/mm^3^ who received ART had substantially lower risk for death than patients with CD4 cell counts ≤200/mm^3^ who were not receiving ART (HR 0.094, 95% CI 0.007–1.22), but this result was not significant.

**Table 3:** Predictors of 49 deaths at 24 months of treatment for 114 HIV-positive and HIV-negative XDR TB patients, KwaZulu-Natal Province, South Africa*

Predictor	No. died/total no. (%)	Univariate analysis			Multivariate analysis†	
		Hazard ratio (95% CI)	p value		Hazard ratio (95% CI)	p value
Sex						
F	27/65 (41.5)	0.88 (0.50–1.54)	0.6484		0.95 (0.51–1.77)	0.8611
M	22/49 (44.9)	1.0 (referent)	NA		1.0 (referent)	NA
Age, y						
<36	25/58 (43.1)	1.03 (0.59–1.80)	0.9285		NA	NA
>36	24/56 (42.9)	1.0 (referent)	NA		NA	NA
Previous TB treatment‡						
Yes	38/92 (41.3)	1.47 (0.53–4.13)	0.4614		1.28 (0.45–3.65)	0.6391
No	4/15 (26.7)	1.0 (referent)	NA		1.0 (referent)	NA
Unknown	7/7 (100)	NA	NA		NA	NA
Initial sputum smear result						
Positive	30/67 (44.8)	1.05 (0.59–1.86)	0.8704		NA	NA
Negative	19/47 (40.4)	1.0 (referent)	NA		NA	NA
HIV status‡						
Positive	36/82 (43.9)	1.14 (0.58–2.25)	0.6971		1.30 (0.61–2.78)	0.4966
Negative	11/25 (44.0)	1.0 (referent)	NA		1.0 (referent)	
Unknown	2/7 (28.6)	NA	NA		NA	NA
Adverse event						
Yes	23/52 (44.2)	1.02 (0.58–1.79)	0.9420		NA	NA
No	26/62 (41.9)	1.0 (referent)	NA		NA	NA

*XDR TB, extensively drug-resistant tuberculosis; NA, not applicable. †Significant variables or variables that caused >10% change in the bivariate hazard ratio were included in the multivariate model. ‡Patients whose HIV status or previous TB treatment history was unknown were excluded from analyses.

**Table 4:** Predictors of 36 deaths at 24 months of treatment for 82 HIV-positive XDR TB patients, KwaZulu-Natal Province, South Africa*

Predictor	No. died/total (%)	Univariate analysis			Multivariate analysis†	
		Hazard ratio (95% CI)	p value		Hazard ratio (95% CI)	p value
Sex						
F	26/52 (50.0)	1.55 (0.75–3.21)	0.2405		1.82 (0.83–4.01)	0.1349
M	10/30 (33.3)	1.0 (referent)	NA		1.0 (referent)	NA
Age, y						
<36	20/45 (44.4)	1.05 (0.55–2.03)	0.8765		NA	NA
>36	16/37 (43.2)	1.0 (referent)	NA		NA	NA
Previous TB treatment‡						
Yes	29/66 (43.9)	1.68 (0.51–5.52)	0.3913		1.70 (0.51–5.65)	0.3865
No	3/12 (25.0)	1.0 (referent)	NA		1.0 (referent)	NA
Unknown	4/4 (100.0)	NA	NA		NA	NA
Initial sputum smear result						
Positive	22/48 (45.8)	1.03 (0.53–2.01)	0.9354		NA	NA
Negative	14/34 (41.2)	1.0 (referent)	NA		NA	NA
Initial CD4 cell count/mm^3^						
<200	13/29 (44.8)	1.02 (0.52–2.02)	0.9495		NA	NA
>200	23/53 (43.4)	1.0 (referent)	NA		NA	NA
Receiving ART						
Yes	18/50 (36.0)	0.54 (0.28–1.03)	0.0633		0.46 (0.22–0.94)	0.0333
No	18/32 (56.3)	1.0 (referent)	NA		1.0 (referent)	NA
Adverse event						
Yes	18/35 (51.4)	1.43 (0.74–2.76)	0.2832		1.89 (0.92–3.86)	0.0812
No	18/47 (38.3)	1.0 (referent)	NA		1.0 (referent)	NA

*XDR TB, extensively drug-resistant tuberculosis; NA, not applicable; ART, antiretroviral therapy. †Significant variables or variables that caused >10% change in the bivariate hazard ratio were included in the multivariate model. ‡Patients whose previous TB treatment history was unknown were excluded from analyses.

Although ART was protective against death among patients co-infected with XDR TB and HIV, it was not associated with sputum culture conversion. After we adjusted for age, sex, ART use, previous TB treatment, adverse drug reactions, and a baseline CD4 cell count ≤200/mm^3^ by using the Cox proportional hazards model, we found that ART use was not associated with culture conversion after 2 months of treatment (HR 0.90, 95% CI 0.23–3.51) or culture conversion during treatment (HR 1.13, 95% CI 0.47–2.7).

When we compared data for HIV co-infected patients hospitalized for initiation of treatment from the first period of the study (December 2006–May 2007) with data of patients hospitalized during the second period (May 2007–November 2007), we found a significant trend toward a higher percentage receiving ART (21/43, 49% vs. 29/39, 74%, respectively; p = 0.02). Multivariate analysis showed that HIV-negative women had higher survival rates than HIV-negative men (HR 0.08, 95% CI 0.01–0.61), Conversely, HIV-positive women had lower survival rates than HIV-positive men (HR 1.82, 95% CI 0.83–4.01), but the difference was not significant.

Data for 109 (96%) patients were analyzed from time of diagnosis. Date of XDR TB diagnosis was unknown for 5 patients. Median time between diagnosis and initiation of therapy was 101 days (IQR 68–150 days). For patients who died, median time between diagnosis and initiation of therapy was 83.5 days (IQR 64–135 days). For patients who survived, median time between diagnosis and initiation of therapy was 118 days (IQR 75.5–163.5 days).

Time to culture conversion appeared to be an insensitive predictor of successful 24-month treatment outcome because for culture conversion at 6 months, sensitivity was only 51% (positive predictive value 85%; negative predictive value 57%) (Table 5). Culture conversion was a better predictor of survival at 24 months because for culture conversion at 6 months, sensitivity was 92% (positive predictive value = 62% and negative predictive value = 97%) (Table 6).

**Table 5:** Sputum culture conversion at intervals of successful treatment for XDR TB patients, KwaZulu-Natal Province, South Africa*

Time from start of treatment, mo	No. cultures converted/total (%)	No. cultures converted/ total converted (%)	Sensitivity, %	Specificity, %	Positive predictive value, %	Negative predictive value, %
1	4/114 (4)	4/42 (10)	5	98	75	43
2	18/114 (16)	18/42 (43)	19	92	78	42
3	29/114 (25)	29/42 (69)	35	88	79	51
4	33/114 (29)	33/42 (79)	42	88	82	53
6	39/114 (34)	39/42 (93)	51	88	85	57
24	42/114 (37)	NA	NA	NA	NA	NA

*XDR TB, extensively drug-resistant tuberculosis; NA, not applicable.

**Table 6:** Sputum culture conversion at intervals of survival for XDR TB patients, KwaZulu-Natal Province, South Africa*

Time from start of treatment, mo	No. cultures converted /total (%)	No. cultures converted/ total converted (%)	Sensitivity, %	Specificity, %	Positive predictive value, %	Negative predictive value, %
1	4/114 (4)	4/42 (10)	12	99	75	79
2	18/114 (16)	18/42 (43)	42	92	61	84
3	29/114 (25)	29/42 (69)	65	86	59	89
4	33/114 (29)	33/42 (79)	77	85	61	93
6	39/114 (34)	39/42 (93)	92	83	62	97
24	42/114 (37)	NA	NA	NA	NA	NA

*XDR TB, extensively drug-resistant tuberculosis; NA, not applicable.

## Discussion

The main findings of our study were a high mortality rate (42%) and a low rate of successful treatment outcomes (22%) for XDR TB patients after completion of 24 months of treatment in a setting with a high incidence of HIV. All deaths in this cohort occurred in the first 12 months after start of treatment. Predictors of deaths in this cohort included TB-specific (TB culture conversion) and HIV-specific (ART use) factors. Consistent with findings in other studies of treatment of drug-resistant TB/HIV, HIV was not independently associated with death (*12*,*13*,*20*). Although HIV was not independently associated with death, use of ART among HIV-infected patients was associated with improved survival. Sex appeared to modify the association between death and HIV because female sex was associated with higher survival rates among HIV-negative XDR TB patients but with higher death rates in women co-infected with HIV than in men co-infected with HIV. However, this finding was not significant in all strata. TB culture conversion was a useful predictor of survival and treatment outcome. However, it was not sufficiently sensitive in this cohort to be a surrogate for successful TB treatment outcome, given the number of patients who ultimately showed treatment failure (n = 7), defaulted (n = 7), or died (n = 4) after TB culture conversion.

Recently, 3 large, randomized, control trials of patients with drug-susceptible TB and co-infected with HIV have been conducted that analyzed different starting points for ART (*21*–*23*). Results of these trials showed that ART started early during TB treatment was associated with improved survival and that most decreases in mortality rates were for patients with low CD4 T-cell counts. As ART use increases among patients co-infected with MDR TB and HIV in KwaZulu-Natal Province, survival among these patients will probably improve. Higher rates of TB culture conversion through more effective drug regimens, including second-generation fluoroquinolones, high-dose isoniazid, and clofazimine, may further improve survival among XDR TB patients (*24*). Complete drug susceptibility testing for all drugs used should be performed at baseline and for XDR TB patients who do not show TB culture conversion after treatment to identify baseline and emergent second-line drug resistance. Furthermore, given that many patients showed reversion to sputum cell cultures positive for TB after initial culture conversion, optimal duration of XDR TB treatment remains unclear, Thus, new regimens, including more potent antimycobacterial agents such as linezolid, TMC207, or new nitromidazoles, may further increase sputum culture conversion rates and survival (*25*,*26*).

Although 4 deaths presumed secondary to capreomycin-associated electrolyte abnormalities occurred early in the study period (*12*), clinicians became more vigilant, tested serum electrolytes more often, and used empiric electrolyte supplementation. Overall, adverse effects were not associated with failure to show sputum culture conversion. Treatment adherence was an unmeasured variable that had a major causal role during treatment for XDR TB and HIV infection. Thus, determining operational methods to measure and improve adherence to ART and second-line and third-line anti-TB drugs is critical for improving outcomes.

Co-infection with drug-resistant TB and HIV has emerged as a major syndemic in South Africa and elsewhere (*26*) and has been comprehensively reviewed (*27*), However, to our knowledge, there are only 2 published studies of early results of treatment for co-infection with XDR TB and HIV. One study, published by our group (*12*), reported low rates of culture conversion (20%) and high mortality rates (42%) after a median of 12 months of treatment for the first 60 consecutive XDR TB patients in the current cohort. The second study, published by Dheda et al. (*13*) from Western Cape Province, South Africa, reported increased mortality rates (36%) and low culture conversion rates (19%) in 174 XDR TB patients after a median follow-up period of 6.9 months after the start of treatment for co-infection with XDR TB and HIV. This cohort had lower but substantial rates of co-infection with XDR TB and HIV and similarly showed no association between HIV and mortality rates for XDR TB patients receiving treatment but a protective effect for ART. There have been several cohort studies of XDR TB in settings with low incidence of HIV, including South Korea, Europe, Peru, and the United States (*28*–*33*). Successful treatment outcomes at 24 months of treatment ranged from 28% to 60%. Only 2 cohorts from Lithuania (*32*) and the United States (*34*) included patients co-infected with HIV.

Limitations to our study included survival bias associated with an observational study at a TB referral hospital. Median time from diagnosis to initiation of treatment for XDR TB was 101 days, which did not decrease over the time of the study. A total of 50%–70% of patients with a diagnosis of MDR TB in KwaZulu-Natal Province had not initiated treatment (*11*,*35*), and those who survived to study referral are probably unique because they were less immunocompromised and had a higher rate of treatment (*11*). We found no association between HIV status and survival or treatment outcome. This result probably reflects countervailing outcomes of patients who received ART and those who did not receive ART. However, this lack of association might be caused by misclassification (refusal to participate in HIV testing), or survival bias.

Because data were collected retrospectively, there were missing data for ART adherence, repeat CD4 T-cell counts, HIV RNA virus loads, adverse events, and details on ART started subsequent to inpatient hospitalization for XDR TB treatment initiation, which may have led to misclassification bias. This result would presumably bias HIV-associated variables toward the null hypothesis. The study was also limited by lack of full TB drug susceptibility testing for capreomycin, PAS, cycloserine, or terizadone to guide treatment choices. Most concerning was the lack of drug-resistance data for capreomycin. In a study published subsequent to our study period, 17 of 19 *M. tuberculosis* isolates from the site of a well-described TB outbreak in KwaZulu-Natal Province were capreomycin resistant (*36*). Because drug susceptibility testing for capreomycin was not available, we may not have identified all cases of XDR TB. Conversely, complete drug susceptibility testing would not have necessarily led to improved regimens because the availability of second-line and third-line anti-TB drugs was limited during this period. In addition, second-generation fluoroquinolones, which may improve outcomes in XDR TB patients, were not available in the public sector for TB treatment during the study (*37**,**38*).

Although not addressed by our study, improvements in treatment outcomes for patients co-infected with MDR TB and HIV will require changes in HIV- and TB-related factors. For HIV, these include more rapid HIV testing for early initiation of ART, appropriate monitoring of CD4 T-cell counts, HIV virus load testing, appropriate opportunistic infection prophylaxis, and improvement in ART adherence. Although not addressed by our study, we recommend that for TB these improvements include widespread implementation of rapid diagnostics, particularly for smear-negative disease; early drug susceptibility testing for first-line and second line agents; improvement in adherence for second-line TB drugs; development of more effective anti-TB drugs and regimens; and guidance of drug selection by timely and ongoing drug susceptibility testing.
